# Electrochemical Skin Conductance as a Marker of Painful Oxaliplatin-Induced Peripheral Neuropathy

**DOI:** 10.1155/2018/1254602

**Published:** 2018-09-27

**Authors:** Jean-Baptiste Delmotte, Abdulkarim Tutakhail, Kahina Abdallah, Pauline Reach, Marguerite D'Ussel, Gael Deplanque, Hélène Beaussier, François Coudoré

**Affiliations:** ^1^Clinical Research Center, Paris Saint Joseph Hospital, Paris, F-75014, France; ^2^CESP/UMR-S 1178, Univ. Paris-Sud, Fac. Pharmacie, INSERM, Université Paris-Saclay, Châtenay-Malabry, F-92290, France; ^3^Neurology Department, Paris Saint Joseph Hospital, Paris, F-75014, France; ^4^Pain Committee, Paris Saint Joseph Hospital, Paris, France, F-75014, France; ^5^Oncology Department, Paris Saint Joseph Hospital, Paris, F-75014, France; ^6^Biology Unit, Paris Saint Joseph Hospital, Paris, F-75014, France

## Abstract

**Purpose:**

Oxaliplatin is a platinum compound widely used in gastrointestinal cancer treatment but produces dose-limiting peripheral neuropathy. New insights into oxaliplatin-induced peripheral neuropathy (OIPN) assessment are needed to detect more effectively this condition. In this context, we conducted Canaloxa study, a prospective preliminary clinical trial that aimed to investigate how Electrochemical Skin Conductance (ESC), a parameter used in small fiber neuropathy assessment, could be helpful in OIPN diagnosis.

**Methods:**

Cancer patients treated for at least three months with oxaliplatin and suffering from clinically OIPN were included. Electrochemical Skin Conductance, thermal thresholds, and neuropathic pain were assessed in all included patients.

**Results:**

During one year, 36 patients were included. The main result was the correlation between ESC and Neuropathic Pain Symptom Inventory score for hands (rho value = -0.69,* p* < 0.0001) and feet (rho value = -0.79,* p* < 0.0001). ESC values were lower in neuropathic patients with painful symptoms than in ones without painful symptoms (*p* = 0.0003 and* p* < 0.0001 for hands and feet, respectively). No correlation was observed between ESC and thermal thresholds.

**Conclusion:**

These preliminary data suggest that ESC could be a useful objective marker of painful oxaliplatin-induced neuropathy and could complement the use of subjective clinical scales. *This study was prospectively registered on clinicaltrials.gov* (NCT02827916) *before patient recruitment has begun*.

## 1. Introduction

Oxaliplatin is a platinum-based cytotoxic drug widely used in oncology since the early 2000s [1]. Its effectiveness is recognized in first-line chemotherapy regimen in metastatic colorectal cancer and in adjuvant therapy of several gastrointestinal cancers [2–5]. However, its use is limited by a development of a disabling sensitive peripheral neuropathy. Oxaliplatin-induced peripheral neuropathy (OIPN) is characterized by a transient cold-induced distal paresthesia and allodynia, sometimes leading to long-term sensory loss and functional impairment [6–8]. OIPN has a negative association with quality of life and this consideration is a major issue in the case of palliative care cancer [9–11]. Correct assessment of oxaliplatin-induced peripheral neuropathy (OIPN) is essential to manage oxaliplatin treatment and therefore limit neuropathy worsening [12].

In clinical practice, OIPN assessment is routinely based on patient-reported outcome and on the National Cancer Institute-Common Terminology Criteria for Adverse Events (NCI-CTCAE) grading scale [13]. Whereas these tests are prevailing for OIPN management, the course of this neuropathy remains unpredictable. While symptoms may resolve after chemotherapy is reduced or discontinued, they can also worsen after discontinuation of the treatment [14]. Therefore, new insights into assessment of OIPN are needed to detect earlier and more effectively this side effect [15, 16].

In clinical research, OIPN assessment is based on small fiber neuropathy (SFN) assessment which requires at least two of the following three examinations: (1) clinical signs of SFN (pinprick and thermal sensory loss and/or allodynia and/or hyperalgesia), (2) abnormal Quantitative Sensory Testing (QST) such as abnormal warm and/or cold thresholds, and (3) reduced epithelial nerve fiber density (ENFD) assessed by skin biopsy [17].

Recently, Saad et al. report potential interest of Sudoscan® technology (Impeto Medical, Paris, France) in OIPN assessment [18]. The Sudoscan device is a quantitative assessment of small fiber neuropathy and the technology is based on Electrochemical Skin Conductance (ESC) measurement in palm and sole [16, 18, 19]. In small fiber neuropathy, sweat glands are underinnervated and sweat function is altered. As a consequence, ESC reflects the density of intraepidermal nerve fibers (IENF): low ESC is related to reduction in IENF density [20].

To further explore the potential interest of using Sudoscan technology in OIPN assessment, we designed a pilot study, Canaloxa, a prospective and monocentric clinical trial. The objective was to study how ESC values were related to other parameters usually employed in OIPN assessment among which thermal thresholds and neuropathic pain scores.

## 2. Materials and Methods

### 2.1. Design of the Study and Regulatory Aspects

Canaloxa study is a prospective, open-label, single group assignment and monocentric clinical trial which was standing at the Department of Oncology at Paris Saint Joseph Hospital (F-75014). This study was prospectively registered at clinicaltrials.gov (NCT02827916) before patient recruitment has begun and all data concerning study design are available online. Canaloxa study was designed as a current care study. Patients included suffered from gastrointestinal cancer at any stages, were treated with oxaliplatin-based regimen for at least 5 cycles (i.e., 10 weeks), and had a clinical neuropathy according to the NCI-CTCAE v4.0 (Common Terminology Criteria for Adverse Events). This standardized scale was used to grade the severity of neurotoxic adverse events with a score from 0 to 5 (e.g., grade-1: paresthesia or loss of deep tendon reflexes; grade-2: moderate symptoms, limiting activity of daily living (ADL); grade-3: severe symptoms, limiting self-care of ADL) [13]. All inclusion criteria are listed in Table 1. The study was conducted according to the Declaration of Helsinki (2013 revision) and obtained approval from the ethics committee of Ile de France II (CPP Ile de France II) on April 2016. This study complied with the recommendations of the International Committee of Medical Journal Editors (ICMJE) on trial registration. Written informed consent was provided to the patients before enrollment. Patients were recruited from April 2016 to March 2017.

### 2.2. Clinical Assessment of Neuropathy

Patients included were assessed for OIPN for only one visit. In addition to the neurological examination based on NCI-CTAE as realized in current practice, OIPN assessment consisted in (1) Electrochemical Skin Conductance assessment using the Sudoscan device (IMPETO Medical, Paris, France) (2) neuropathic pain assessment using the Neuropathic Pain Symptom Inventory (NPSI) scale and (3) thermal sensory assessment using the MSA thermal stimulator (SOMEDIC AB, Hörby, Sweden).

Before assessment, patients remained in an air-conditioned room (temperature controlled: 22°C ± 2°C) for at least 15 minutes in order to be in stable conditions of blood flow and skin temperature. All the tests were performed by a single trained technician.

#### 2.2.1. Electrochemical Skin Conductance Assessment

Electrochemical Skin Conductances were measured with the Sudoscan device (IMPETO Medical, Paris, France). Sudoscan technology results in a quantitative assessment of small fiber neuropathy. On the technical side, an electrochemical reaction between chloride ions of the sweat and the electrodes produces a current related to the sweat glands innervated by small fibers [19, 21].

Patients were asked to firmly apply bare hands without jewelry and bare feet to the electrodes during the measurement period. The data were displayed on a monitor in the form of a diagram and four ESC values (right and left hands; right and left feet). High conductance (ESC > 60 *μ*Siemens) is related to a normal sweat function (i.e., no neuropathy) whereas low conductance (ESC < 40 *μ*Siemens) reveals a dysfunction of the sweat function (advanced peripheral neuropathy). ESC between 40 and 60 *μ*Siemens represent first signs of peripheral neuropathy. Mean ESC between left and right was calculated to produce a single ESC value for the two hands and a single ESC value for the two feet for each patient. The operator was previously trained with a series of 20 healthy volunteers.

#### 2.2.2. Neuropathic Pain Assessment

Neuropathic pain symptoms were recorded, and their severity were scored using the NPSI, a 11-point (0-10) numerical scale of ten neuropathic symptoms (burning, squeezing, pressure, electric shocks, stabbing, pain evoked by a brush, by pressure or by cold, tingling, and pins and needles). The total NPSI score, i.e., the sum of individual subscores out of 100, was calculated [22]. This questionnaire has already been used in previous studies dealing with OIPN [6].

#### 2.2.3. Thermal Sensory Assessment

Thermal sensory assessment consisted of measuring four parameters using the MSA thermal stimulator (SOMEDIC AB, Hörby, Sweden): cold detection threshold (CDT), warm detection threshold (WDT), cold pain threshold (CPT), and heat pain threshold (HPT). Thermal stimuli were applied using a Peltier probe (25 mm wide and 50 mm high) applied to the skin of the thenar eminence and to the skin of the sole. The probe was set at a baseline temperature of 32°C and stimulus was delivered at 1°C/s in both directions to a defined and stable value. Thresholds measurement was based on the method of limits [23]. For warm and cold detection thresholds, patients were instructed to indicate by clicking on a mouse as soon as they felt a change in temperature. Mean thresholds were calculated using three consecutive measurements, spaced by a random period of 4-10 seconds. Detection thresholds (CDT, WDT) were presented as a temperature variation, relatively to 32°C (basal temperature) whereas pain thresholds (CPT, HPT) were presented in absolute values. To obtain reproducible and comparable results, instructions given to the patients were standardized and the operator was previously trained with a series of 20 healthy volunteers. In contrast to ESC, normal values of thermal sensitivity thresholds do not reach consensus [24]. Reference thresholds may vary according to the device settings, patient characteristics and environment [23, 25].

### 2.3. Statistical Analysis

Results were presented as mean ± standard deviation (SD) or standard error of the mean (SEM). As these data are known to have nonnormal distribution, comparisons tests were based on Wilcoxon tests and correlation tests were based on Spearman test [20]. Receiver operating characteristics (ROC) curves were calculated to compare the utility of ESC in painful OIPN diagnosis. Area under the curve higher than 0.7 was considered to have a good sensitivity and specificity [26].

Statistical analyses were performed with R software, v.64 bits 3.4.1, and* p* < 0.05 was considered to be significant.

## 3. Results

From April 2016 to March 2017, 36 patients were included (18 women and 18 men). The mean age of patients was 64 ± 11 years (range: 26-84). Patient's cancer was predominantly colorectal (n = 19, 52.8%) and at metastatic stage (stage IV) (n = 20, 55.5%). Neuropathy was predominantly at grade I (n = 33, 91.6%) according NCI-CTCAE v4 scale (Table 2).

### 3.1. Electrochemical Conductivity of the Skin (ESC)

**Figure 1:**
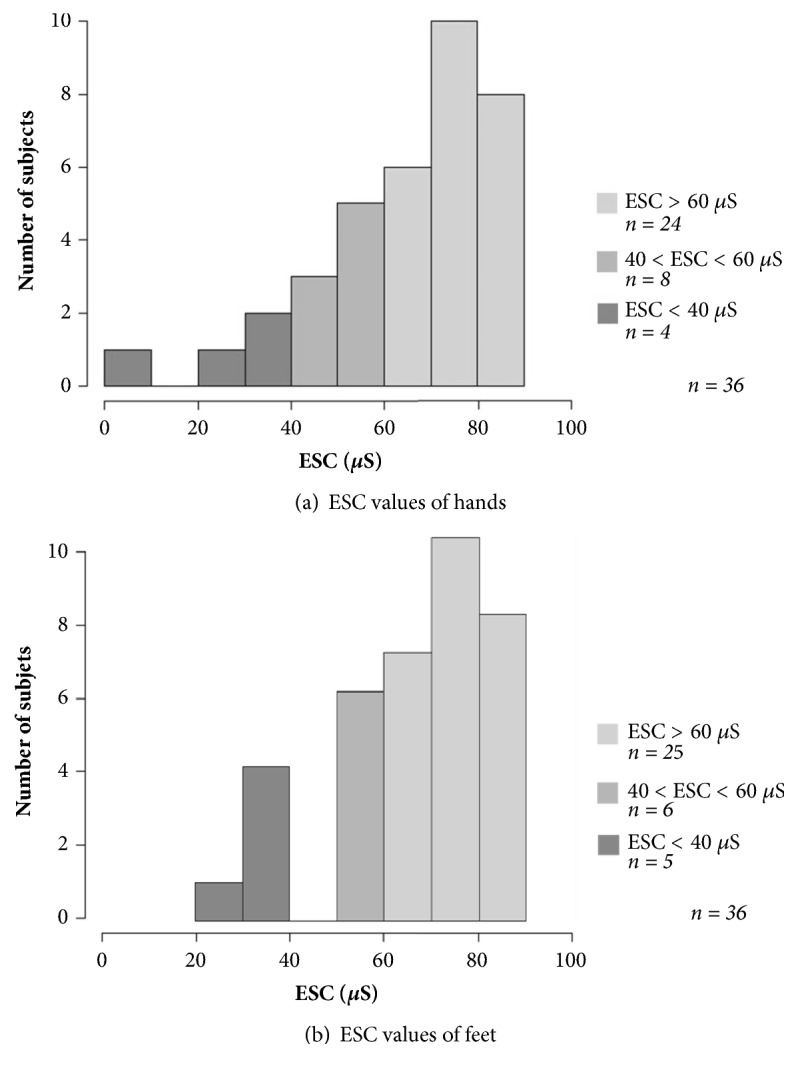
Distribution of the ESC values measured on hands (a) and feet (b) of oxaliplatin-treated patients (n = 36). High conductance values (ESC > 60 *μ*S): no dysfunction of the sweat function. Intermediate conductance values (40-60 *μ*S): first signs of a possible peripheral autonomic neuropathy. Low conductance values (ESC < 40 *μ*S): dysfunction of the sweat function and advanced peripheral neuropathy. ESC: Electrochemical Skin Conductance. *μ*s: microSiemens.

Mean ESC values were similar between hands and feet: 65.3 ± 19.0 *μ*S and 65.3 ± 16.6 *μ*S, respectively. No relationships between ESC and sex, age, or body mass index were highlighted for hands (*p* = 0.51,* p* = 0.32,* p* = 0.50, respectively) and feet (*p* = 0.25,* p* = 0.75, and* p* = 0.53, respectively). Low conductances (ESC < 40 *μ*S) were detected for 4 patients on hands and 5 patients on feet, whereas intermediate values (40 *μ*S < ESC < 60 *μ*S) were detected for 8 patients (hands) and 6 patients (feet) (Figures 1(a) and 1(b)). Normal conductances (ESC > 60 *μ*S) were detected for 24 patients (hands) and 25 patients (feet). ESC values did not have a normal distribution (*p* = 0.01 and* p* = 0.008 for hands and feet, respectively, Shapiro-Wilk test).

### 3.2. Neuropathic Pain

Regarding neuropathic pain assessed with NPSI questionnaire, pain was declared by 55.5% (20/36) of oxaliplatin-treated patients (Table 3(a)). According to NCI-CTCAE, 51.5% (17/33) of patients with a grade 1 neuropathy and 100% (3/3) with a grade 2 had pain (*p* = 0.23, Fisher exact test). Among them, 90% (18/20) of patients said they felt pain caused by a cold object; 70% and 45% reported pain as prickling and tingling, respectively. One patient declared a pain symptom as electric discharges.

**Figure 2:**
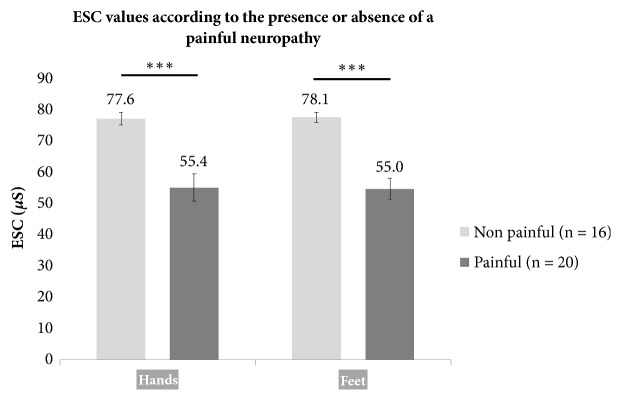
**ESC values measured on hands and feet according to the presence or absence of a painful neuropathy.** ESC values were lower in neuropathic patients with painful symptoms than in patients without painful symptoms: 55.4 ± 19.7* vs* 77.6 ± 7.9 *μ*S (*p* = 0.0003) and 55.0 ± 15.0* vs* 78.1 ± 6.6 *μ*S (*p < *0.0001) for hands and feet, respectively.

**Figure 3:**
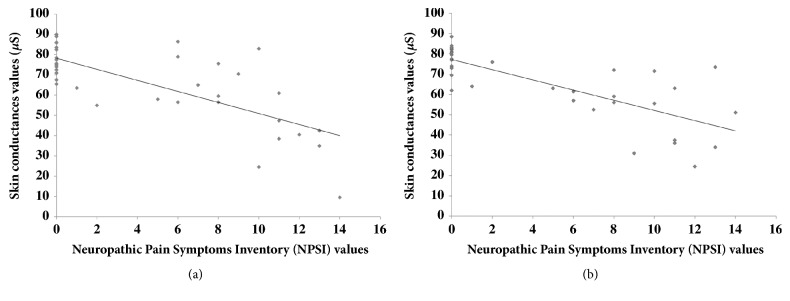
Correlation graphs between Electrochemical Conductance (ESC) in *μ*Siemens (*μ*S) of hands (a) and feet (b) of treated patients and Neuropathic Pain Symptom Inventory (NPSI) scores (n = 36). ESC are correlated with NPSI score for hands (rho = -0.69,* p* < 0.0001) and feet (rho value = -0.79,* p *< 0.0001).

Regarding the relationship between ESC and the presence of a painful neuropathy, ESC values were lower in neuropathic patients with painful symptoms than in patients without painful symptoms: 55.4 ± 19.7 *μ*S* vs* 77.6 ± 7.9 *μ*S (*p* = 0.0003) and 55.0 ± 15.0 *μ*S* vs* 78.1 ± 6.6 *μ*S (*p < *0.0001) for hands and feet, respectively, (Figure 2). Furthermore, ESC were correlated with NPSI score for hands (rho = -0.69,* p* < 0.0001) and feet (rho = -0.79,* p* < 0.0001) (Figures 3(a) and 3(b)).

**Figure 4:**
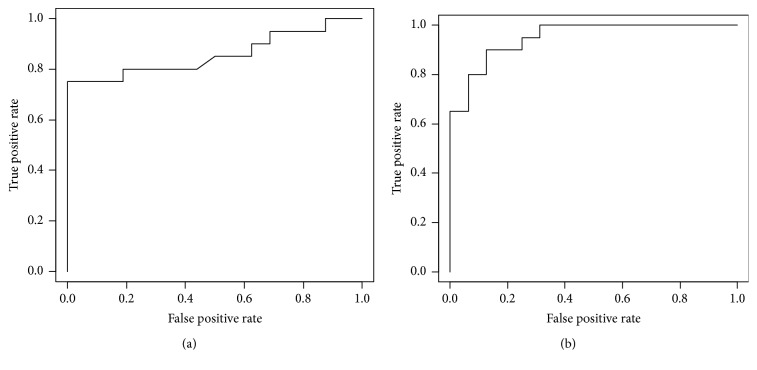
**Receiver operating characteristics (ROC) curves for ESC of hands (a) and feet (b) using a positive NPSI score as reference.** AUC = 0,86 for (a) and AUC = 0,95 for (b). (20 patients with painful symptoms and 16 patients without painful symptoms).

Performance of the Sudoscan device to detect a painful OIPN was estimated with receiver operating characteristics (ROC) curves. Positive NPSI score was used as a reference for the ROC curves of hands and feet ESC (Figure 4). Areas under the ROC curve were employed to assess the performance of the device in term of sensibility and specificity. Areas under the curve were 0.86 and 0.95, for hands and feet, respectively, higher than 0.7, thus showing a good sensitivity and specificity [26].

### 3.3. Thermal Thresholds

Results are presented in Tables 3(b) and 4. No correlation was found between both detection and pain thresholds (CDT, WDT, CPT, and HPT) and ESC for hands and feet.

## 4. Discussion

The aim of this work was to explore oxaliplatin-induced peripheral neuropathy with the Sudoscan device, a recent technology based on Electrochemical Skin Conductance measurement and already used in diabetic-induced peripheral neuropathy diagnosis. The onset of OIPN remaining unpredictable, new insights into OIPN assessment are needed to detect and prevent more effectively this side effect. In this context, Sudoscan is being assessed to test its opportunity in OIPN diagnosis [18].

In Canaloxa study, pathological Electrochemical Skin Conductance (ESC) was detected for approximately one-third of neuropathic patients with a mean ESC value of 65.3 ± 19.0 *μ*S for hands and 65.3 ± 16.6 *μ*S for feet. These figures are consistent with the few first ones published for oxaliplatin-treated patients. In Saad et al. study, ESC measured in oxaliplatin-treated patients were 63 ± 2 *μ*S in hands and 66 ± 3 *μ*S in feet. ESC values are known to have a nonnormal distribution, as confirmed by a Shapiro-Wilk test, which made us choose nonparametric tests such as Spearman correlation test and Wilcoxon test. Novak asserts that ESC values can be normalized with weight [20]. The main result of this study is the correlation between the ESC values (hands and feet) and the NPSI score. The ESC was diminished in neuropathic patients with painful symptoms compared to ones without painful symptoms. This relationship was associated with a high performance of the Sudoscan device to diagnose painful OIPN. ROC curve analysis showed significant results for both hands and feet ESC. It is interesting to notice that ESC is fully objective whereas NPSI score is fully subjective. In the same way, there was a relationship between ESC and another subjective score, the Total Neuropathy Score (TNSc), in oxaliplatin-treated patients [18]. In Novak et al. study, ESC failed to correlate with NPSI score [20]. However, in this study, ESC was adjusted for weight and the population was different, with no chemotherapy-induced peripheral neuropathy. Interestingly, when adjusted for weight, our normalized ESC values have a normal distribution (*p* = 0.942 for hands and* p *= 0.14 for feet, Shapiro-Wilk test). However, these normalized ESC no longer correlate with NPSI score (rho values = -0.36 and -0.42 for hands and feet, respectively). According to Novak, ESC could be affected by subjects' weight because of the variation of pressure on the stainless steel electrodes, but concomitant studies showed no obvious correlation between ESC and weight [20, 27]. Facing this inconsistency, it cannot be excluded that there is a little influence of weight on ESC. Such a minimal influence may perhaps reduce an existing difference between patients with low ESC/high NPSI and patients with high ESC/low NPSI resulting in a reduction of the statistical tests' power after normalization. A study with more patients could bring a response to that question. According to the manufacturer, interpretation of ESC values is based on raw values, what has been respected here.

Platinum derivatives are already known to impair neurite outgrowth [28]. Nerve damage was thereafter confirmed by clinical studies using human skin biopsies [29] and* in vitro* studies [30]. ESC is diminished in patients who have a reduced number of IENF and this reduction is proportional to the density of fibers [20]. Compared to skin biopsies, the interest of Sudoscan device is its noninvasive nature. As ESC was diminished in our OIPN patients with painful symptoms, this study suggests that oxaliplatin could impair small nerve fibers. Nerve cells damage caused by oxaliplatin would be responsible for the pain of OIPN. However, cancer or vitamins deficiency can cause subclinical neuropathy, so we cannot assert that oxaliplatin is exclusively responsible for the nerve impairment [29, 31].

It should be noticed that two-thirds of the neuropathic patients had normal ESC. We can wonder whether (1) ESC was not sensitive enough for OIPN diagnosis or whether (2) ESC can only diagnose painful OIPN. Regarding sensitivity of Sudoscan device to detect OIPN, it should be noticed that, in many neurotoxic processes such as diabetic neuropathy, vegetative disorders are of late onset. By analogy, normal ESC in neuropathic patients could be explained by the delayed onset of vegetative oxaliplatin-neuropathy [32]. This may indicate that oxaliplatin toxic process during the first three months of treatment would not be sufficient to induce impairment of sweat gland function although all patients experiment clinical neuropathy, according to the NCI-CTCAE. There is potentially a poor sensitivity of this method in OIPN detection. A recent meta-analysis dealing with the reliability of the ESC to measure sudomotor or sensory nerve fiber function has questioned this test. According to Rajan et al., large combined data sets of the studies included in the meta-analysis do not support a high sensitivity and specificity. Besides, the pathological thresholds of ESC could depend on pathophysiological parameters and ethnicity [33]. It is obvious that further studies taking into account these concerns could clarify the question. Regarding painful neuropathy detection, low ESC were measured in patients in pain essentially, but, in Saad et al. study, low ESC were measured in neuropathic patients more generally [18]. We can also wonder whether the suitable neurological fibers were explored. Indeed, the Sudoscan device does not explore larger sizes fibers (*e.g.* A-delta). It might be relevant to perform in a future work a nerve conduction study (NCS) to assess large fibers conduction and to better understand whether ESC may be influenced in part by dysfunction of these large fibers in oxaliplatin-treated patients. Indeed, in diabetic subjects, ESC correlates with NCS [34].

Thermal thresholds measured were consistent with those published in previous studies dealing with OIPN [7]. Thermal thresholds in oxaliplatin-treated patients seemed to be greater than in nontreated patients that suggest the onset of a small fiber pathology in these patients [25, 32]. This consideration could reveal a hypoesthesia to thermal stimuli that impair with treatment. No correlation was observed between ESC values and thermal detection or pain thresholds. This suggests that the Sudoscan device and the MSA thermal stimulator assess different nerve fibers function. The Sudoscan device assesses amyelinic sympathetic C-fibers which innervate the sweat glands while the MSA thermal stimulator assess myelinated A-delta fibers and unmyelinated C-fibers. More precisely, warmth receptors are thought to be unmyelinated C-fibers, while those responding to cold have both C-fibers and thinly myelinated A-delta fibers [35]. A painful thermal stimulus is mediated by C-fibers. The poor sensitivity of thermal thresholds to diagnose small fiber neuropathy was already reported [36, 37]. Reproducibility is low and pain thresholds are even more dispersed than detection thresholds [38]. As a matter of fact, interpretation of thermal thresholds is a little bit tender. Thermal thresholds depend on many psychological and physiological, innate and acquired factors. Environmental conditions, instructions given by the manipulator, patient motivation, and ethnic origin may also contribute to the variability of the results [23]. So, some physiological elements, like the variation of the thickness of the* stratum corneum*, may have introduced measurement biases in the thermal thresholds measurement, which could explain the absence of correlation between the different tests.

Canaloxa study was a preliminary study with a limited number of patients and with one-day assessment. This pilot study was conducted to investigate the feasibility of the Sudoscan device to detect OIPN before performing a longitudinal and controlled study with a larger number of patients. ESC values collected in this study provided information about ESC distribution and standard deviation, essential information for the design of the future study. A control group of cancer-matched patients, not treated with oxaliplatin, and a longitudinal study with ESC values measured before treatment are necessary to attribute the neurological impairment to oxaliplatin. Moreover, regarding non-inclusion criteria, it could be relevant to perform a screening for autonomic symptoms and to dose folates and B12 vitamins at inclusion.

## 5. Conclusion

In conclusion, this study suggests that painful OIPN could be detected using the Sudoscan device. Electrochemical Skin Conductance measurement is a promising, easy-to-perform, and noninvasive test that could be done in current care practice to help the clinician in OIPN management. Otherwise, this study toughens the comprehension of the pathophysiology of OIPN in the field of small fiber neuropathy. However, Canaloxa was a feasibility study and these results have to be confirmed by a longitudinal and controlled study with in a greater number of patients.

## Figures and Tables

**Table 1:** Eligibility criteria of Canaloxa study.

**Inclusion criteria**
Patient from the Department of Oncology at Paris Saint Joseph Hospital, male or female, aged over 18.
Patient suffering from any type of cancer, at any stages (estimated according to the TNM classification)
Patients treated with oxaliplatin based regimen for at least 5 cycles (*i.e.,* 10 weeks)
Patients with clinical neuropathy objectified according to the NCI-CTCAE v4.0

**Non-inclusion criteria**

Patient with brain or leptomeningeal metastasis
Patient previously treated with cisplatin
Patient addicted to alcohol
Diabetic patient (based on fasting glucose)
Patient receiving calcium or magnesium salts intravenously
Patients with implantable medical devices (pacemakers, implantable defibrillators)
Patient suffering from psychiatric disorders
Patient treated with at least one of the following drug (active in neuropathic pain relieve): venlafaxin, carbamazepin, gabapentin, pregabalin, clomipramin, amitriptylin, imipramin, and duloxetin.

**Table 2:** Characteristics of patients included in Canaloxa study between April 2016 and March 2017 in the Oncology department of Groupe Hospitalier Paris Saint Joseph (GHPSJ).

	**Patients**	
	**n**	**(**%**)**
**Gender**		
**Woman**	18	50
**Man**	18	50

**Age (years)**		
**< 50**	4	11 .11
[**50-60[**	6	16.66
[**60-70[**	14	38.88
**> 70**	12	33.33

**Location of the tumor**		
**Colon**	12	33.33
**Stomach**	6	16.66
**Liver**	1	2.77
**Pancreas**	9	25
**Rectum**	7	19.44
**Peritoneum**	1	2.77

**Cancer Stage**		
**I**	0	0
**II**	1	2.77
**III**	15	41.66
**IV**	20	55.55

**Chemotherapy protocol**		
**FOLFOX**	26	72.22
**FOLFIRINOX**	9	25
**GEMOX**	1	2.77

**Grade of neuropathy according to NCI-CTCAE v4.0**		
**I**	33	91.66
**II**	3	8.33

**Table:** (a) *Neuropathic Pain Assessment.* (1) Number of patients without neuropathic pain (null NPSI score). (2) Number of patients with neuropathic pain (positive NPSI score). (3-5) Number of patients with positive response at questions 10, 11 and 12. NPSI: Neuropathic Pain Symptoms Inventory.

**Neuropathic Pain Assessment**	***n***	%
**(1) NPSI score = 0 **	16	44.4
**(2) NPSI score > 0**	20	55.5
**(3) Pain caused by the contact with a cold object (Q10)**	18	90
**(4) Tingling (Q11)**	9	45
**(5) Swarming (Q12)**	14	70

**Table:** (b) *Thermal Sensory Assessment.* Thermal thresholds were measured with the MSA thermal stimulator on hands and feet (n = 36 patients): (i) Cold and Warm Detection Thresholds (CDT, WDT) are presented as relative values (expressed as variations of temperature from the basal temperature of 32°C). (ii) Cold and Heat Pain Thresholds (CPT, HPT) are presented as absolute temperature values.

**Thermal Sensory Assessment**	**Hands (M ±ET)**	**Feet (M ±ET)**
**CDT (**Δ°**C)**	- 3.4 ± 2.2	- 7.0 ± 4.0
**WDT (**Δ°**C)**	2.9 ± 2.0	11.6 ± 4.4
**CPT (**°**C)**	13.6 ± 6.7	12.8 ± 6.8
**HPT (**°**C)**	45.6 ± 4.1	48.6 ± 4.6

**Table 4:** Matrices of correlation crossing ESC values and thermal thresholds. Spearman tests were performed. CDT, WDT: Cold and Warm Detection Thresholds. CPT, HPT: Cold and Heat Pain Thresholds. ESC: Electrochemical Skin Conductance.

	ESC of hands		ESC of feet	
	rho	*p*	rho	*p*
CDT (hands, feet)	-0.007	0.97	0.18	0.28
WDT (hands, feet)	0.20	0.25	-0.14	0.40
CPT (hands, feet)	0.00	0.99	-0.07	0.69
HPT (hands, feet)	0.14	0.42	-0.13	0.47

## Data Availability

The data used to support the findings of this study are available from the corresponding author upon request.

## Additional Points

Electrochemical Skin Conductance correlates with neuropathic pain symptoms inventory score in a 3-months oxaliplatin-treated patients (n = 36) but does not correlate with thermal sensitivity tests.

## References

[1] Graham J., Muhsin M., Kirkpatrick P. (2004). Oxaliplatin. *Nature Reviews Drug Discovery*.

[2] André T., Boni C., Navarro M. (2009). Improved overall survival with oxaliplatin, fluorouracil, and leucovorin as adjuvant treatment in stage II or III colon cancer in the MOSAIC trial. *Journal of Clinical Oncology*.

[3] Haller D. G., Tabernero J., Maroun J. (2011). Capecitabine plus oxaliplatin compared with fluorouracil and folinic acid as adjuvant therapy for stage III colon cancer. *Journal of Clinical Oncology*.

[4] Xu W., Kuang M., Gong Y., Cao C., Chen J., Tang C. (2016). Survival benefit and safety of the combinations of FOLFOXIRI ± bevacizumab versus the combinations of FOLFIRI ± bevacizumab as first-line treatment for unresectable metastatic colorectal cancer: A meta-analysis. *OncoTargets and Therapy*.

[5] Raymond E., Faivre S., Woynarowski J. M., Chaney S. G. (1998). Oxaliplatin: Mechanism of action and antineoplastic activity. *Seminars in Oncology*.

[6] Attal N., Bouhassira D., Gautron M. (2009). Thermal hyperalgesia as a marker of oxaliplatin neurotoxicity: A prospective quantified sensory assessment study. *PAIN*.

[7] Binder A., Stengel M., Maag R. (2007). Pain in oxaliplatin-induced neuropathy - Sensitisation in the peripheral and central nociceptive system. *European Journal of Cancer*.

[8] Kokotis P., Schmelz M., Kostouros E., Karandreas N., Dimopoulos M.-A. (2016). Oxaliplatin-Induced neuropathy: a long-term clinical and neurophysiologic follow-up study. *Clinical Colorectal Cancer*.

[9] Mols F., Beijers T., Vreugdenhil G., Van De Poll-Franse L. (2014). Chemotherapy-induced peripheral neuropathy and its association with quality of life: A systematic review. *Supportive Care in Cancer*.

[10] Stefansson M., Nygren P. (2016). Oxaliplatin added to fluoropyrimidine for adjuvant treatment of colorectal cancer is associated with long-term impairment of peripheral nerve sensory function and quality of life. *Acta Oncologica*.

[11] Tofthagen C., Donovan K. A., Morgan M. A., Shibata D., Yeh Y. (2013). Oxaliplatin-induced peripheral neuropathy’s effects on health-related quality of life of colorectal cancer survivors. *Supportive Care in Cancer*.

[12] Saif M. W., Reardon J. (2005). Management of oxaliplatin-induced peripheral neuropathy. *Therapeutics and Clinical Risk Management*.

[13] National Cancer Institute Common Terminology Criteria for Adverse Events (CTCAE) Version 4.0. http://www.cancer.gov/.

[14] Trivedi M. S., Hershman D. L., Crew K. D. (2015). Management of Chemotherapy-induced peripheral neuropathy. *American Journal of Hematology / Oncology®*.

[15] Calvet J., Dupin J., Winiecki H., Schwarz P. (2013). Assessment of small fiber neuropathy through a quick, simple and non invasive method in a german diabetes outpatient clinic. *Experimental and Clinical Endocrinology & Diabetes*.

[16] Smith A. G., Lessard M., Reyna S., Doudova M., Singleton J. R. (2014). The diagnostic utility of Sudoscan for distal symmetric peripheral neuropathy. *Journal of Diabetes and its Complications*.

[17] Devigili G., Tugnoli V., Penza P. (2008). The diagnostic criteria for small fibre neuropathy: from symptoms to neuropathology. *Brain*.

[18] Saad M., Psimaras D., Tafani C. (2016). Quick, non-invasive and quantitative assessment of small fiber neuropathy in patients receiving chemotherapy. *Journal of Neuro-Oncology*.

[19] Casellini C. M., Parson H. K., Richardson M. S., Nevoret M. L., Vinik A. I. (2013). Sudoscan, a noninvasive tool for detecting diabetic small fiber neuropathy and autonomic dysfunction. *Diabetes Technology & Therapeutics*.

[20] Novak P. (2016). Electrochemical skin conductance correlates with skin nerve fiber density. *Frontiers in Aging Neuroscience*.

[21] Freedman B. I., Smith S. C., Bagwell B. M., Xu J., Bowden D. W., Divers J. (2015). Electrochemical skin conductance in diabetic kidney disease. *American Journal of Nephrology*.

[22] Bouhassira D., Attal N., Fermanian J. (2004). Development and validation of the Neuropathic Pain Symptom Inventory. *PAIN*.

[23] Shy M. E., Frohman E. M., So Y. T. (2003). Quantitative sensory testing: report of the therapeutics and technology assessment subcommittee of the American academy of neurology. *Neurology*.

[24] Rolke R., Baron R., Maier C. (2006). Quantitative sensory testing in the German research network on neuropathic pain (DFNS): standardized protocol and reference values. *PAIN*.

[25] CHOO JCSKK (1998). Temperature sensitivity of the body surface over the life span. *Somatosensory & Motor Research*.

[26] Mandrekar J. N. (2010). Receiver operating characteristic curve in diagnostic test assessment. *Journal of Thoracic Oncology*.

[27] Vinik A. I., Smith A. G., Singleton J. R. (2016). Normative values for electrochemical skin conductances and impact of ethnicity on quantitative assessment of sudomotor function. *Diabetes Technology & Therapeutics*.

[28] Luo F. R., Wyrick S. D., Chaney S. G. (1999). Comparative neurotoxicity of oxaliplatin, ormaplatin, and their biotransformation products utilizing a rat dorsal root ganglia in vitro explant culture model. *Cancer Chemotherapy and Pharmacology*.

[29] Koskinen M. J., Kautio A.-L., Haanpää M. L. (2011). Intraepidermal nerve fibre density in cancer patients receiving adjuvant chemotherapy. *Anticancer Reseach*.

[30] Massicot F., Hache G., David L. (2013). P2X7 cell death receptor activation and mitochondrial impairment in oxaliplatin-induced apoptosis and neuronal injury: cellular mechanisms and in vivo approach. *PLoS ONE*.

[31] Kosturakis A. K., He Z., Li Y. (2014). Subclinical peripheral neuropathy in patients with multiple myeloma before chemotherapy is correlated with decreased fingertip innervation density. *Journal of Clinical Oncology*.

[32] Park S. B., Lin C. S. Y., Krishnan A. V., Goldstein D., Friedlander M. L., Kiernan M. C. (2011). Long-term neuropathy after oxaliplatin treatment: challenging the dictum of reversibility. *The Oncologist*.

[33] Rajan S., Campagnolo M., Callaghan B., Gibbons C. H. (2018). Sudomotor function testing by electrochemical skin conductance: does it really measure sudomotor function?. *Clinical Autonomic Research*.

[34] Selvarajah D., Cash T., Davies J. (2015). SUDOSCAN: a simple, rapid, and objective method with potential for screening for diabetic peripheral neuropathy. *PLoS ONE*.

[35] Darian-Smith I., Johnson K. O., LaMotte C., Shigenaga Y., Kenins P., Champness P. (1979). Warm fibers innervating palmar and digital skin of the monkey: Responses to thermal stimuli. *Journal of Neurophysiology*.

[36] Lefaucheur J.-P., Wahab A., Planté-Bordeneuve V. (2015). Diagnosis of small fiber neuropathy: A comparative study of five neurophysiological tests. *Neurophysiologie Clinique / Clinical Neurophysiology*.

[37] Tobin K., Giuliani M. J., Lacomis D. (1999). Comparison of different modalities for detection of small fiber neuropathy. *Clinical Neurophysiology*.

[38] Tin S. N. W., Andrade D. C. d., Goujon C., Planté-Bordeneuve V., Créange A., Lefaucheur J.-P. (2014). Sensory correlates of pain in peripheral neuropathies. *Clinical Neurophysiology*.

